# Developing a strong sustainability research program in marketing

**DOI:** 10.1007/s13162-020-00185-6

**Published:** 2020-12-10

**Authors:** Melea Press

**Affiliations:** University of Glasgow, University Avenue, Glasgow, G12 8QQ UK

**Keywords:** Consumer culture theory, Critical marketing, Increasing impact, Marketing strategy, Paradigm shift, Research program, Strong sustainability

## Abstract

This article takes stock of sustainability research in marketing and argues for developing a Strong Sustainability Research (SSR) program, led by a Consumer Culture Theory (CCT) approach. First, I define weak vs. strong sustainability and identify two main problems with continuing to research business with the weak sustainability approach. Second, I discuss past approaches to sustainability research in marketing, which primarily promote weak sustainability. Third, I use the agriculture industry to illustrate how an SSR program in marketing could be developed to bring insights to practitioners and policy makers and build new modes of production, consumption and exchange. Finally, I suggest that the SSR program facilitates collaboration between mainstream marketing and CCT researchers by providing a common ontological platform that can transform epistemological differences into complementary strengths. I argue SSR is a way that marketing research can gain broad impact and relevance.

## Introduction

Recently, there have been several calls to address the type and level of impact and the relevance of marketing research. These calls have been directed at mainstream marketing research (MMR; Clark et al. 2014; Hunt 2018), Consumer Culture Theory (CCT; Thompson 2019), and the *Journal of Marketing* audience (MacInnis et al. 2020) more generally. These critiques address issues of methodology (Clark et al. 2014), relevance for audiences beyond marketing academics (Clark et al. 2014; McDonagh and Prothero 2014; MacInnis et al. 2020), and the “dominant theoretical and analytical vernacular of marketing research and practice” (Thompson 2019).

However, in the many calls for making marketing research more relevant, the need to directly address sustainability issues, that is, to build strategy for a globalized market facing the effects of climate change, has been overlooked. This includes insights for navigating emerging challenges in natural resource procurement (Mikdashi 2019; Rees 2017) and protection (Tompkins and Adger 2004; George et al. 2015, 2018), a decreased appetite for pollution (Fink 2020) and lack of accountability (Gray 2006), increased political and social instability (Levy et al. 2017; Hartley et al. 2017), and the resulting risk throughout the supply chain (Ghadge et al. 2020). The current body of work on sustainability in marketing has begun to address several aspects of these sustainability issues in individual articles and past and forthcoming special issues (Kemper and Ballantine 2019; McDonagh and Prothero 2014; White et al. 2019; e.g. *Industrial Marketing Management, Journal of Marketing, Journal of Marketing Management, Journal of Macromarketing, Journal of the Academy of Marketing Science)*. However, much of this work is focused on the trees, or micro-level issues and outcomes (White et al. 2019), rather than the forest, or contextualized systemic issues and outcomes. More research is imperative to create intellectual material addressing sustainability issues that can be used to innovate and support new market and marketing strategy (Flyvbjerg 2005) and inform public policy.

In this article I identify opportunities for marketing research to increase its impact with regard to sustainability. I clarify the differences between weak and strong sustainability and argue that marketing researchers must engage with strong sustainability to address current industrial and practitioner issues, and to bring renewed interest and relevance to marketing research. I argue that a CCT approach fits well to the complexities of Strong Sustainability Research (SSR). I identify five activity streams to develop an SSR program in marketing, which I illustrate with an example from the agriculture industry. Finally, I point to opportunities for both mainstream marketing and CCT researchers in the SSR project.

## Sustainability and the business domain

Sustainability and what it means for businesses, markets, the planet earth, its inhabitants and natural systems, is arguably the most pressing issue of this moment. We start the new decade with dire scientific predictions about human activities and their consequences for the future of this planet (Lenton et al. 2019). The somewhat benign-sounding term “climate change” is now more appropriately described as a climate crisis, as effects of climate change are already disastrous for many ecosystems, economies, communities and individuals (IPCC n.d.). Academic studies and reports in the media show the dramatic effects of the climate crisis, including the wildfires in Australia (Sullivan 2020) and the western United States, the increasing strength, speed and frequency of hurricanes in the Atlantic Ocean, the dying coral reefs (Morrison et al. 2019) and ocean acidification, and species collapse (IPBES 2019), and some evidence indicates that global pandemic Covid-19, which has reached over 47 million cases and over 1.2 million deaths worldwide, as of early November 2020, and brought social and economic disaster to individuals and countries, is a result of habitat disruptions (Goudarzi 2020). For the moment, tensions between “business” and “sustainability” are present in daily life (Hsiang et al. 2017), the dichotomy of economic productivity vs. human health and safety is a political battleground, social distancing emerges as a privilege, and the price of oil dropped below zero for the first time.

In this moment of dichotomies, we see the extreme politicization of the climate crisis (Taplin 2020) and growing resistance to participation in decreasing climate effects as evidenced by the withdrawal of the US from the Paris Climate Accord (Johnson 2019), the destruction of the Amazon Rainforest (Dwyer 2019), and weakening climate policies (Popovich et al. 2019). At the same time, 193 countries have adopted the United Nation’s 17 Sustainable Development Goals (SDGs) to “achieve a better and more sustainable future for all” (UN, n.d.), firms are arguing for more and less (depending on the firm) stringent environmental standards (Tabuchi 2019), and investors are starting to use indicators of effects of the climate crisis in company risk assessments (Fink 2020). The issue of sustainability is acknowledged as important across environmental, social and economic systems; now is the time to revise the way we approach sustainability research in marketing to address contemporary needs, and to increase relevance and impact.

The Consumer Culture Theoretics (CCT) approach to research is well equipped to engage a new conversation around sustainability issues that managers, consumers, policy makers, industry leaders, and governments are facing; issues that will continue to increase in complexity and intensity. In this article, I identify how a CCT approach can contribute key insights to sustainability research, suggest a sustainability research program for marketing to deliver new conceptualizations and insights to managers and policy makers, and identify ways that mainstream marketing research (MMR) and CCT can contribute and collaborate in this project.

## Weak and strong sustainability

The main definitional difference between weak sustainability (WS) and strong sustainability (SS) is how one views ecosystem services. WS asserts that natural capital and manufactured capital can substituted for each other, while SS rejects such substitutability and argues some functions can only be performed by natural capital in the ecosystem and thus should be given special protection (Dietz and Neumayer 2007). WS promotes growth-based capitalism and is reflected in the rhetoric of the business case for sustainability, the idea that sustainability activities will improve the firm’s bottom line by improving reputation and building customer loyalty (Bonini and Swartz 2014; Kotler 2011; Laszlo and Cescau 2017; Lenz et al. 2017; Kemper and Ballantine 2019; Lovins et al. 1999; Lubin and Esty 2010; Nidumolu et al. 2009, Whelan and Fink 2016). WS is micro-focused and explores individual elements of economic activity and its impact separately rather than as pieces of an integrated whole, while SS is contextualized and integrated across actors and stakeholders (Roome 2012). While definitional differences between WS and SS can be easily identified, the implications of adopting weak vs. strong sustainability in our research perspectives are more challenging, and so far have been largely avoided (with a few notable exceptions, c.f. Gray 2010; TEEB (The Economics of Ecosystems and Biodiversity) n.d.).

## The problem with weak sustainability

Weak sustainability emerged in the 1970s as a way to account for non-renewable natural resources as a factor of production, essentially extending the neoclassical theory of economic growth (Dietz and Neumayer 2007; Hartwick 1978; Solow 1986). WS treats natural capital and human-made capital as perfectly substitutable resources (Gutés 1996) and allows for products of economic growth to compensate for the loss of natural resources and ecosystem services (Gray 2010). That is, WS adopts the notion that something (e.g. an economy) is sustainable if the total stock of resources does not diminish over time (Pearce and Atkinson 1993). This approach to sustainability aligns well with the growth-based capitalist system (Hartwick 1978; O’Riordan 1989; Solow 1993). In WS, firms maintain their goals to increase sales and grow profits as consumption increases.

WS is reflected in research in marketing that tends to be micro-focused (impact of x on y) and constrained to variables (DVs and IVs) rather than cultural, social, or historic contexts. For example, Karmarkar and Bollinger (2015) explore the effects of reusable shopping bags on consumer purchases and find that using reusable bags increases the purchase of organic and indulgent foods. Luo and Bhattacharya (2006) ask whether CSR affects the market value of a firm and find that the effect is mediated by customer satisfaction and moderated by corporate capabilities. In some papers, responsibility for sustainability is placed on consumers, as shown by Giesler and Veresiu (2014), while key benefits from sustainability are focused on firm profit by generating consumer value (Kotler 2011) rather than on creating shared value (Porter and Kramer 2011). Governments are given a role to play “if consumers and voters push them” (Kotler 2011, 134), but are not seen as an integral part of markets or economic exchange. The result is disjointed micro-level knowledge about firms and consumers, with firms benefitting from WS while other institutions are marginalized.

There are two main problems with the WS view. The first is that it does not specifically acknowledge, and cannot specifically address the complex strategic challenges that firms are currently facing. I will come back to this with the industrial example below. The second problem is that even as firms have embraced sustainability as evidenced by the dramatic increase in sustainability reporting (GRI 2017; Higgins et al. 2018), the natural environment and human quality of life have continued to rapidly decline (BSR 2015; Borowy 2014; Dyllick and Muff 2016; Landrum 2018; Visser 2010). There are different ideas about why this is the case, despite the great corporate effort presumed by the increased reporting. Some authors argue that research into sustainability across business disciplines has focused on reducing unsustainability (Ehrenfeld 2012; Málovics et al. 2008), rather than on creating sustainability (Gray 2006; Gray and Milne 2002; Milne et al. 2006) or increasing capacities in the system (Sen 2005). Others note that there is inadequate understanding about what corporate sustainability means (Gladwin et al. 1995; Shrivastava 1995a, 1995b), and thus research has focused on clarifying the business case for sustainability through measurement activities (Gray 2010; Landrum 2018). Still others identify that the focus on measurement of mainstream business metrics has categorically excluded larger environmental and social justice issues (Banerjee 2008; Ehrenfeld 2012). Finally, researchers have identified the lack of integration of micro and macro-level understandings of sustainability (Dyllick and Muff 2016; Landrum 2018). Milne et al. (2006) show that when businesses adopt the metaphor of sustainability as a journey, it allows them to focus on minor improvements and micro-processes (rather than substantive issues) that are reflected in corporate activities and reporting (Gray 2010) and contribute to a misleading corporate narrative (Cho et al. 2015; Russo and Harrison 2005). The focus on de-contextualized specific problems, instead of implications for social and environmental systems that stem from those problems, normalizes WS and the associated epistemologies found in industrial sustainability engagement and academic research (Higgins et al. 2018; Parguel et al. 2011). While such explanations for the continued environmental and human degradation may identify areas for new research, they do not provide decision support, nor do they identify insights to address substantive issues of sustainability (Flyvbjerg 2005). Further, these explanations point to the need for research to identify business strategies and practices that could address the issues such critiques raise by creating new models for strategy development and market exchange that would positively impact quality of life and the natural environment.

## Strong sustainability

*Strong sustainability* (SS) does not view the products of economic growth as acceptable compensations for the loss of natural resources and ecosystem functions (Dresner 2002; Gutés 1996; Jones et al. 2008; Peattie and Peattie 2009; Sandberg and Polsa 2015). Enacting SS would require a dramatic reassessment of the growth-based capitalist system (Gray 2010; Kilbourne et al. 2002; McDonagh and Prothero 2014; Shultz and Holbrook 1999), which seems untenable without exploring alternate possibilities for exchange practices. Further, ignoring that what happens in the natural world affects the social and economic world avoids our responsibility as researchers (Tadajewski 2018b), and goes directly against mainstream definitions of marketing, including “creating, communicating, delivering, and exchanging offerings that have value for customers, clients, partners, and society at large” (AMA n.d.).

I suggest we need a strong sustainability research (SSR) program in marketing that addresses issues and tensions associated with strong sustainability (Landrum 2018; Upward and Jones 2015). Several frameworks for sustainability exist, such as the very broad United Nations (UN) Sustainable Development Goals (SDGs; UN n.d.) which presents 17 broad goals that cross disciplines, literatures, and various reporting guidelines. On the other end of the spectrum lies the narrow Global Reporting Initiative (GRI), which addresses industry-specific issues and remains focused on reporting techniques (Cho et al. 2015; Russo and Harrison 2005). However, neither provides insight into the historically and culturally situated context of industrial, social and market activity that firms are engaged in, as a CCT-led SSR program in marketing could do.

Other business fields have started to address what it would mean for business functions to view the economic system as a subsystem of the biosphere (Costanza and Daly 1992; Daly 1990, 2015; Georgescu-Roegen 1975), and recognize that sustainable activities must respect the interdependency of systems, species, and biophysical constraints over time and space (Stål and Bonnedahl 2016). Articles in top journals in accounting (Gray 2006; Gray 2010; Milne and Gray 2013; Cooper and Owen 2007) and management identify what SS would look like across firm activities (Rangan et al. 2015; Porter and Kramer 2011; Starik and Marcus 2000) and across supply chains (Shrivastava 1995a, 1995b). These articles highlight the need to work with the complex relationships of influence between individual firms and ecological systems (Gray 2010) and signal that there is stomach, even curiosity and desire, to explore what this paradigmatic shift (Kuhn 1962) in perspective from WS to SS might look like. It is time for marketing research to begin a cohesive effort to address issues and tensions associated with SS.

## The current state of sustainability research in marketing

Reviews of the state of sustainability research in marketing use bibliographic analyses and critical syntheses (Chabowski et al. 2011; Harper and Peattie 2011; Kemper and Ballantine 2019; Leonidou and Leonidou 2011; McDonagh and Prothero 2014). McDonagh and Prothero (2014) provide a broad review of sustainability work in marketing, including articles published in 13 different journals. One striking takeaway across these reviews is that sustainability work in marketing is constrained by “conservatism and disciplinary rigidity… and inward-looking tendencies amongst marketing scholars” (Harper and Peattie 2011) and that “mainstream journals… consider, for the most part, the managerial implications of micro, environmental questions, without paying enough attention to the macro relationships between marketing and the natural environment” (McDonagh and Prothero 2014, 1203). Sustainable marketing articles debate what sustainable marketing is (Hunt 2011; Kemper and Ballantine 2019; McDonagh and Prothero 2014) yet fail to address what it looks like as an immediate substantive issue for the businesses, institutions, organizations, and individuals doing the marketing. These articles often discuss sustainability in terms of a journey and a set of marketing strategies, but they tend to lack a clear definition of what sustainability means or looks like at the end of such a journey (Higgins et al. 2018; Milne et al. 2006).

I will now discuss several different approaches to studying sustainability in marketing; key elements are summarized in Table 1 The first is an MMR approach. Mainstream marketing sustainability work has largely explored sustainable consumption (White et al. 2019), predictors of sustainable consumption (Kotler 2011; Menon and Menon 1997; Mick 2006; Paharia 2020; White et al. 2012; Winterich et al. 2019), and issues of how sustainability activities affect firm performance. MMR articles tend to gloss over definitions of sustainability (White et al. 2019; Chabowski et al. 2011), focusing instead on micro-aspects of sustainability and neglecting a larger picture (Giesler and Fischer 2017). Generally, “sustainability” articles published in *Journal of Marketing* in the past 10 years address consumer topics such as how thinking about recyclables turning into new products increases recycling behaviors (Winterich et al. 2019) and how using reusable grocery bags affects in-store behavior (Karmarkar and Bollinger 2015). Managerial-focused sustainability articles in *JM* signal benefits for businesses that can adapt to the “urgent demand for sustainability” (White et al. 2019, 23; Banerjee et al. 2003), and state that firms that both operate sustainably and consider new business models that encourage sustainable consumption reap even more long-term profits (Kotler et al. 2010; White et al. 2019). These articles also address topics such as how corporate social responsibility activities relate to firm performance (Kang et al. 2016) and consumer attitudes to the corporation (Lichtenstein et al. 2004), and whether social initiatives can be used to create positive brand associations (Simmons and Becker-Olsen 2006; White et al. 2012). Overall, these articles treat micro-aspects of sustainability as independent variables and the research questions in these articles ask what is the effect of X (a sustainability variable) on Y (a consumer or firm outcome) (Karmarkar and Bollinger 2015; Paharia 2020; Winterich et al. 2019). These articles primarily reference other MMR articles, positioning their papers against the largely psychological phenomena they address. MMR sustainability work does not make space for higher level marketing strategy, business models, or supply chain issues, and it tends to hide critical environmental and social issues in global markets (Tadajewski 2014; McDonagh and Prothero 2014; Peattie 2001; Prothero et al. 2011; Rangan et al. 2015).

**Table 1:** Comparing approaches to research on sustainability in marketing

	MMR	CCT	Critical	SSR
Approach	Weak sustainability	Some examples of strong sustainability	Questions weak sustainability	Strong sustainability
How do we talk about sustainability? What is true about sustainability and business?	• The main objective of business is profit • Sustainability is a journey (lacking a defined destination) • Sustainability is about measurement and performance • The economy supports the other two elements of sustainability – environment, society	• Exchange is context-dependent • Narrow and broad contexts matter • Sustainability is a journey (lacking a defined destination) • Sustainability is complex, networked and nuanced • The three elements of sustainability – environment, society and economy – are equally important	• The current business models we use cause the current problems we have • Markets as provisioning systems are not sustainable • Benefits of the market are created at the expense of others (in other current or future societies) or the environment • Current exchange models, practices and beliefs are the reason for our current sustainability problems	• Sustainable exchange considers multiple systems and systemic interactions • Critical engagement with externalities from exchange • The biosphere is the base upon which society and the economy are created • Value is recognized in terms of co-evolving interdependencies across stakeholders • Sustainability is a biosphere enriching system
Typical research topics/questions	• How does sustainability as an independent variable affect consumer behavior? • How does sustainability as an independent variable affect firm outcomes?	• What is sustainable marketing? • How can firms and consumers engage in more sustainable behavior? • How do contextual constraints affect sustainable business practice? • How do consumers experience and engage in sustainable consumption? • How is responsible consumption conceptualized? • How do consumption practices form and change?	• Exploring macro-level constructs to explain marketing, markets and behavior • Questioning assumed relationships between DSP and sustainable outcomes • Consumption within the capitalist economy is inherently non-sustainable • Conceptualizing consumption in a macro-level context	• How do elements of a sustainable economy function? • How does business tie into a sustainable economy? • How do production and consumption support a sustainable economy? • How can business activities be embedded in a broader framework of sustainable resource exchange? • How do markets function to support sustainable system? • How do industries and social systems integrate to create capability-enhancing value?
Level of analysis	• Individual • Firm	• Individual • Group/consumption community • Firm • Industry • Living (biological) actors	• Individual • Country	• Individual • Firm • Industry • Institutional actors (government, NGOs, policy makers) • Community • Living (biological) actors
Goal	• Measure and report effects of sustainability activities on firm profits • Measure and report effects of sustainability activities on consumer behavior	• Framework development • Unpack individual engagement with sustainable choices • Understand consumption behavior in context	• Problematize accepted norms, practices, and ideologies as constraints to sustainability	• Develop modalities for sustainable exchange • Unpack contextual constraints at the consumer, firm, industry, and supply chain level • Increase sustainability for multiple stakeholders
Stance toward sustainability	• Independent variable	• Mixed: Tangential, indirectly addressed or glossed-over; also contextualized, integrated across actors	• Critical, direct	• Integral to entire research plan

The second approach to sustainability research is macromarketing, which has a long history of addressing issues around marketing and the environment (Fisk 1973, 1974), the role of markets and market actors (Dobscha and Ozanne 2001; Kilbourne et al. 1997; Shultz and Holbrook 1999), and the role of sustainability for marketing functions (Arvidsson 2008; Kilbourne 2004; Prothero et al. 2010). Marcomarketing research is itself divided into a Development School that sees markets and marketing as tools for social development and human welfare, and a Critical School that questions the consequences of markets and marketing (Mittelstaedt et al. 2014). The Development School adopts a WS lens to address quality of life and market issues. This school addresses similar research topics to MMR, including attitudes to recycling (Pelton et al. 1993) and marketing strategy issues (Mitchell et al. 2010). It also addresses broader themes such as structural and institutional challenges to marketing (Lewin et al. 2011) and the effect of political regulation, access to capital and labeling systems on organic food diffusion (Thøgersen 2010). Similar to MMR research, these articles often insert the sustainability dimension as an independent variable.

The Critical School questions the WS lens and promotes critical examination of structures, institutions and practices that affect outcomes (Mittelstaedt and Kilbourne 2008; Shultz 2007; Kilbourne et al. 1997; Kilbourne 2004). This includes exploring presentations of the dominant economic growth-based paradigm (Kilbourne et al. 1997) in film (McDonagh and Brereton 2010) and commodity discourses (Prothero et al. 2010), the tradeoffs implicit in anthropocentrism (Kadirov 2011) and how we could rethink marketing effectiveness (Varey 2012). This approach to sustainability research critiques existing norms, ideologies, structures and assumptions, but it offers few concrete paths forward to explore and create new ones. One exception is Dolan’s (2002) article, which argues for consumer behavior to be viewed as a manifestation of historical social and cultural activities, that is, as the micro-level materialization of macro-level processes.

The third approach is found in articles that seek to develop sustainability research by benchmarking what has been done, for example using complex models of citations to identify research areas for sustainability in marketing (Chabowski et al. 2011), or adding variables to mainstream ideas such as the “market-oriented sustainability framework” (Crittenden et al. 2011) and the “market orientation plus” framework (Hult 2011). These articles are largely incremental in their contributions, and add additional variables to existing frameworks, and tend to be theoretical, rather than empirical.

The fourth element in this list is not an approach to sustainability research, but an illustration of missed opportunities and may indicate a general lack of understanding of sustainability in our field. For example, in a commentary on marketing strategy, Varadarajan (2018) compares the way trees interact with their environment to capture and use resources with the way firms should interact with their environment, making direct connections to product line proliferation. Yet, while this is a direct naturalist metaphor, it fails to include critical elements of the natural system within which the trees exist. That is, the comparison does not acknowledge the limited resources available to trees in the natural environment, how their activity changes the environment, or how they contribute to or shape the environment (Kohn 2013; Simard et al. 1997). Including these elements in the comparison would change the assumptions about appropriate long-term product strategy. These omissions, or lack of engagement with sustainability, reflect business school blinders, neoliberal market ideology blinders (Fitchett et al. 2014), and general preferences of the field of marketing to include convenient elements of sustainability, but not engage with the issue as a whole.

The last genre of sustainability work I will discuss is a CCT approach to research. Many of these articles are found outside top journals and identify contextual issues that constrain consumers’ ability to act (Press and Arnould 2009), and identify economic, cultural and structural issues that may be at play (Earley 2014; Moisander et al. 2009; Kilbourne 2004). Some of these articles address issues of social justice, inequality, and the unfair distribution of the effects of the climate crisis (Askegaard and Linnet 2011; Kilbourne 2004). Others explore how sustainable practices develop (Denegri-Knott, Nixon and Abraham 2018) and address macro-level aspects of sustainable consumption (de Burgh-Woodman and King 2013; Kjellberg 2008) including how sustainable-oriented changes in food consumption depend on choices of material and immaterial actors across markets and society (D'Antone and Spencer 2015; Cherrier 2009). These articles identify context-specific consumption patterns and connect to macro socio-cultural themes.

CCT articles that address sustainability issues in top journals tend to be positioned in terms of more established themes and are not explicit in their sustainability orientation. For example, Giesler and Veresiu (2014) use data collected at the World Economic Forum, a meeting of leaders from business, politics, academia and society to “improve the state of the world” (weforum.org), to discuss, among other things, sustainable development. However, their article is positioned in terms of the creation of the consumer subject and consumer responsibilization. Press et al.’s (2014) article explores how ideology affects firms’ strategic orientation and uses data from organic commodity agriculture. It refers to issues of soil health, loss of topsoil, health issues associated with chemical farming, and logistic and ideological constraints to making more sustainable choices. However, rather than position explicitly as a sustainability paper, it aligns with MMR market orientation literature. Sheth’s (2011) article asks how emerging markets could inform marketing thought and offers a brief critique of the colonialist perspective often brought to research on emerging markets. He identifies five dimensions of emerging markets and connects them to MMR marketing theory, strategy, policy, and practice. He engages with sustainability in his article in several meaningful ways, but also omits “sustainability” in the abstract and framing. These articles are integrative in terms of research approach and findings. However, as examples of CCT sustainability research, their impact could be increased if they were easier to identify as part of a growing body of work on sustainability.

The overall impact of these different approaches to sustainability research is limited by the insular way that research is developed, pursued, and reported. Each approach is isolated, in part because of the self-serving ways they develop and position their research questions, and how they refer to their own bodies of research, frequently not reaching across literatures even within the field of marketing. Because there is little cross-talk, there has not emerged a comprehensive research stream on sustainability in marketing, but rather several approaches with different assumptions, ontologies, and levels of analyses making a disjointed body of work that is difficult to compare or build on (see Table 1). Finally, each approach to research could work in an SS perspective and the SSR framework below is meant to facilitate this goal, however, with the exception of the critical and CCT approaches, past research on sustainability in marketing has tacitly adopted the WS perspective. This puts sustainability research in marketing in a lagging role in identifying, conceptualizing, and shaping knowledge about sustainability. We have an opportunity to build a body of intellectual insight that can inform strong sustainability policy and practices.

## CCT’s relevance to SSR

An SSR program must operate at two different levels. The first level is immediate practical support to changing industrial practices, which I illustrate below. Building on its rich history of nuanced insights into various elements of marketing strategy, a CCT approach is well-equipped to address pressing sustainability issues in consumption, industry and markets (Arnould et al. 2019). CCT allows for contextualized insights across marketing topics including building business models for shifting brand, product and customer strategy (Peñaloza and Venkatesh 2006; Cayla and Arnould 2013; Etimur and Coskuner-Balli 2015), unpacking how industry and firm legitimacy are created and enacted in new contexts (Humphreys 2010; Press and Arnould 2011b), identifying sticking points in developing market orientation in new markets (Press et al. 2014; Gebhardt et al. 2006), and exploring new market dynamics (Giesler 2003, 2008; Martin and Schouten 2013; Karababa and Ger 2011; Scaraboto and Fischer 2013). All of these topics require additional investigation focused on navigating the effects of the climate crisis.

The second level of the SSR program is a higher-level conceptualization of modes of exchange. The higher-level goal of this research program must address the role of key social, economic and environmental stakeholders in exchange practices and identify new mental and practical models for how exchange can increase, not just profits for shareholders, but efficacy and vitality for all individuals, communities, and the natural world. It explores sustainable business practices as those that increase the capacities of organisms (e.g. participants, stakeholders, especially humans) in the ecosystem, and increase their access to resources that allow them to realize those capacities (Sen 2001, 2005).

The higher level goals of the SSR program will require shifts in assumptions about research problems (MacInnis et al. 2020) and conceptualizations of models of exchange (see Table 2). It will require broad inclusion across layers of social fabrics and reconsideration of the moral responsibility of governments and business (Bouchet 2017; Smith 1776), and necessitate rethinking value creation as a culturally-informed (Karababa and Kjeldgaard 2014) and contextually situated (Askegaard and Linnet 2011) process that takes into account the nonmarket institutions that support the market economy (Sen 2001). Further, it will require researchers to explicitly address who and what can, should and does create and benefit from “value.” The higher-level of the SSR program requires that researchers consider consumption as the result of long and complex socio-cultural processes and not merely as the individual choice or responsibility (Campbell et al. 2013), and that, rather than focus on opposites (e.g. such as degrowth and anticonsumption; Hobson 2013), we reimagine what production and consumption look like as interconnected capacity-building processes (e.g. symbiosis, mutually-enriching relationships, interconnected systems; Kohn 2013; Simard 2018; Stamets 2005).

**Table 2:** Current and SSR marketing research focus

	Current research conceptualization	SSR focused conceptualization
Approach	Static, bounded	Dynamic, integrating
Unit of analysis	Individual (consumer, manager, firm)	Networked actors
Assumptions	• The main objective of business is profit • Human-made capital is substitutable for natural capital • Sustainability is good for business • The three elements of sustainability (environment, society and economy) are equally important • Value is created through human use • A thing becomes a resource when its value is recognized by humans	• Exchange systems create value • There is no substitute for natural capital • Effects of sustainability are assessed at micro, meso and macro levels • The natural environment must be healthy for social and economic activity to thrive • Value is created in interdependencies between the human-nonhuman world • Resources are historically and socially contingent
Conceptual dynamism	• Static decision context and time orientation • Business and consumer behavior are understood based in terms of economic utility and individual choice • Value creation is predicated on human superiority over material systems	• Dynamic decision context explored across time • Business and consumer behavior are understood as culturally, socially and historically embedded practices • Value creation is predicated on an interdependent understanding of resources
Data collection	• Respondents selected based on convenience • Focus on response number, not appropriateness of respondents • Externalities not considered in respondent/informant selection	• Multiple in-situ respondents • Identify appropriate informants based focal phenomena • Reflexivity in informant inclusion and exclusion • Integrate data on living (biological) actors as system stakeholders
Model of exchange	• Capitalist growth model • Ownership • Promoting wealth for business owners through material consumption • Filling consumer needs/wants through consumption activities • Production and consumption deplete resources	• Interdependent model • Capability increasing and enhancing • Creating value for living beings and systems through exchange • Consumer wants are reflections of larger systems • Production, exchange, and consumption support SDGs

Building on its rich history of nuanced insights into various elements of marketing strategy, a CCT approach can address these issues of integration and specificity in SSR (Arnould et al. 2019). Thus a CCT approach can help generate insight into creating new business models for shifting brand, product and customer strategy (Peñaloza and Venkatesh 2006; Cayla and Arnould 2013; Etimur and Coskuner-Balli 2015) with increasingly constrained resources, or how industry and firm legitimacy are created and enacted in a context where norms are rapidly changing (Humphreys 2010; Press and Arnould 2011b). A CCT approach can further identify sticking points in developing market orientation for an operating environment with new challenges (Press et al. 2014; Gebhardt et al. 2006), and shed light on new market dynamics (Giesler 2003, 2008; Karababa and Ger 2011) and the role of consumers in innovation (Martin and Schouten 2014; Scaraboto and Fischer 2013).

## Building a strong sustainability research program

An SSR program in marketing is an opportunity to bring marketing academics into the critical work of developing a world where consumption and exchange practices themselves build greater social justice and improve the natural environment, and where we recognize that all actors operate within the natural system of planet earth (Lenton et al. 2019; Dresner 2002). We need to measure value creation not only in terms of profits to shareholders, but also in terms of contributions to the wellbeing of human and non-human stakeholders and the ecosystems of which we/they are a part. We must recognize that institutions and governments provide a backbone of justice, governance, basic education, health, safety and infrastructure, and support innovation and creativity (Bouchet 2017; Smith 1776). We must acknowledge that firm activity happens within dynamic markets (Araujo 2007; Giesler and Fischer 2017; Giesler 2008) in macro systems (Askegaard and Linnett 2011; Clark et al. 2014; Ulrich 1993). We must, further, explicitly acknowledge that none of our social or economic systems can exist without a functional natural environment (McDonagh and Prothero 2014; Lenton et al. 2019) and recognize humans as part of a deeply interconnected ecosystem (Gray 2010). The SSR program identifies activities we can start right now in our research and engagement with practitioners, industry, policy-makers, and other stakeholders to address urgent and growing sustainability issues.

**Fig. 1:**
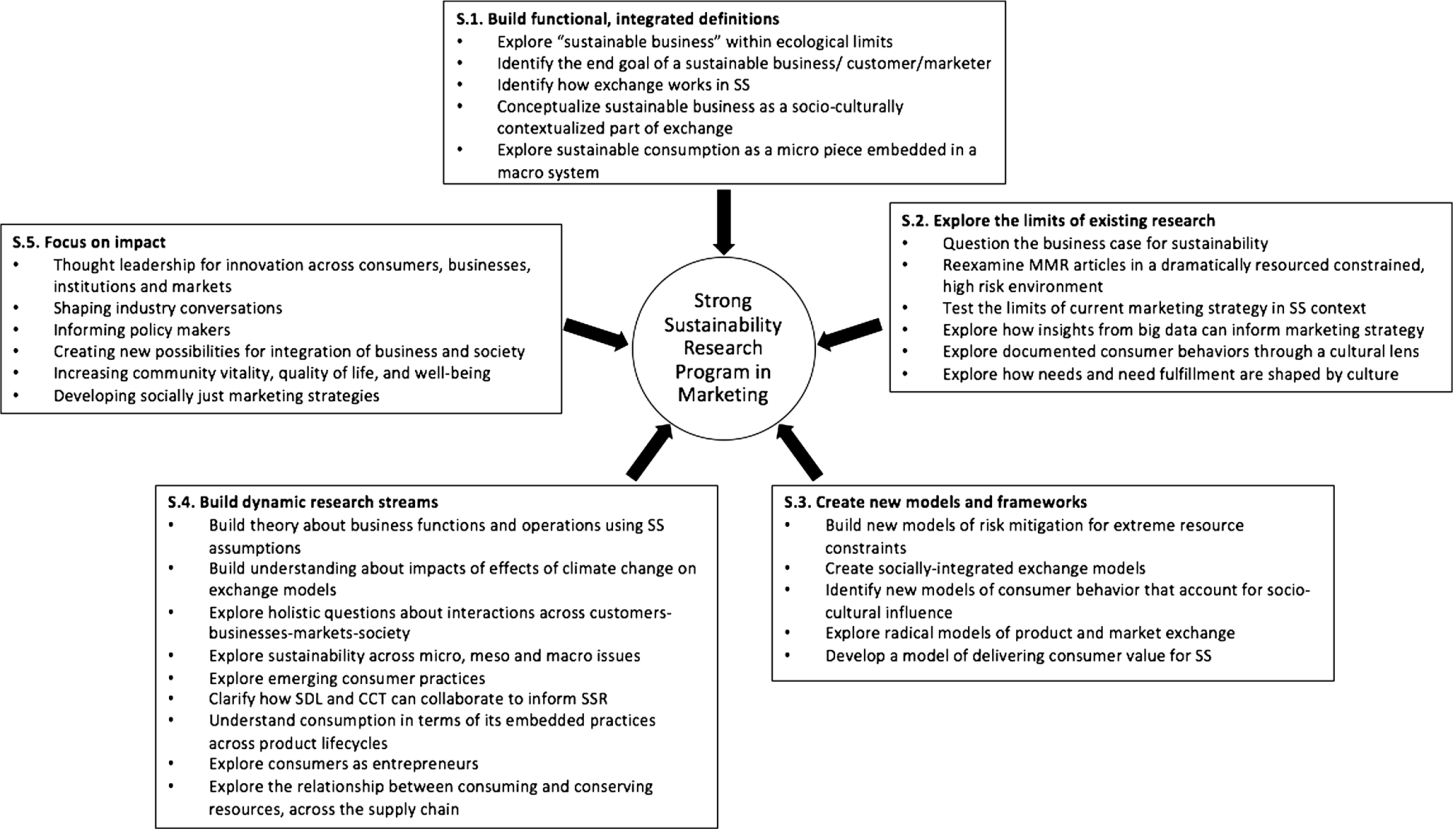
Five activity streams for a Strong Sustainability Research program in marketing

I suggest five activity streams for developing an SSR program in marketing; these are summarized in Fig. 1. These activity streams build on previous marketing research to create a holistic program that addresses current and future issues faced by organizations and society as a result of the effects of the climate crisis, increased political and social instability, a changing relationship with the natural world, decreased availability of natural resources, changing customer preferences, and increasing inequality. In other words, this program will directly address issues and tensions associated with SS. I will now introduce the agriculture industry as a context for SSR and then illustrate the five streams of the SSR program using the example of the agriculture industry.

### The agriculture industry

Agriculture is a complex and diverse industry that reaches into food production, hospitality, tourism, and also textile and paper production. Early marketing strategy was built from agricultural production and exchange, and it remains a core global concern. Issues of sustainability in agriculture become more urgent as global population increases, soil fertility decreases, weather patterns become more unpredictable, and local climates change. The agricultural industry is most susceptible to the climate crisis, which is already negatively affecting production in tropical climates and is predicted to worsen (Verchot et al. 2007; Solomon et al. 2017). The cascading effects that the climate crisis sets in motion directly impact agricultural productivity, producers, agricultural markets, and associated markets (Mendelsohn and Neumann 2004; Brown et al. 2017). Farmers are already facing challenges associated with the climate crisis, such as increasingly extreme and unpredictable weather leading to increased pests and disease (Deutsch et al. 2018). Historically, long periods of drought, famine and lack of water lead to social disintegration and disappearance, as with the Anasazi in the twelfth century (Benson et al. 2007), and more recently, to climate migration and political instability, and war, as in Syria (Gleick 2014).

The agricultural industry needs insight into new models of innovation, relationship management and customer procurement, and branding (Ertimur and Coskuner-Balli 2015; Martin and Schouten 2014) to build resilience into their businesses (c.f., Press et al. 2014; Thompson and Coskuner-Balli 2007; Weber et al. 2008), reduce risks and increase positive externalities across their supply chains (c.f., Martin et al. 2019; D'Antone and Spencer 2015). There are many examples of agricultural firms engaging in sustainability activities for the purposes of reducing their own risks. For example, McCain invests in local potato growers and partners with small sustainable entrepreneurs to gain legitimacy in local markets (Press et al. 2020), Mars and Hershey engage in activities to increase productivity and market information transparency in cacao grower regions in hopes of securing their supply of cacao (Bonini and Swartz 2014) and Coca-Cola closely monitors its water use and impact, especially after numerous scandals around polluting sources (Whelan and Fink 2016; Earth Talk 2019) and decimating local water supplies (Agerholm 2017; Patsiaouras et al. 2015). However, most of these “new” strategies are actually strategies to remain viable in the growth-based economic system that led to the current issues, with firms trying to shore up their status quo, and small entrepreneurs exploring new strategies. Hypermarkets and traditional food retailing is declining and firms across the agriculture industry are challenged to identify new strategies (Herbert et al. 2018).

### Agriculture and SSR

I select agriculture as an industrial example to illustrate SSR because there is already a large body of CCT work addressing sustainable agriculture (SA) that explores the culturally, socially and historically embedded practices of SA production, consumption and exchange (Batat et al. 2016; Chaudhury and Albinsson 2014; Giordano et al. 2018; Herbert et al. 2018; Mars and Schau 2017, 2018; Press and Arnould 2011a, 2011b; Thompson and Coskuner-Balli 2007; Press and Arnould 2014; Smith Maguire et al. 2017; Thompson and Press 2014; Visconti et al. 2014; Watson and Ekici 2017). It tends to look at SA as an alternative to the big agriculture/corporate food system (Thompson and Coskuner-Balli 2007; Giordano et al. 2018) that builds personal connections (Mars and Schau 2017) and counteracts feelings of risk and helplessness (Press and Arnould 2011a; Thompson and Coskuner-Balli 2007) and explores local food as expressing “ideals of civic agriculture and just sustainability” (Visconti et al. 2014, 4).

Within the agriculture industry lies a blurred line between pastoral agriculture and industrial agriculture that is rendered fuzzier still by conflicting ideologies, economic assumptions and social goals, all with implications for sustainability. Decades of research across social and hard science critique agricultural practices for their negative impact on ecosystems and human health (Carson 1964), and SA entrepreneurs have emerged as counteracting those industrial forces (Thompson and Coskuner-Balli 2007). By approaching this industry with the intention of contributing to SSR in marketing, scholars can continue to expand relevance and focus from small entrepreneurs to the industry as a whole, and build a body of work that supports policy decisions, industry actions and helps create new market models. As such, an SSR program focusing on the agriculture industry must adopt SS assumptions (see Table 2), and seek answers to SS questions about how agriculture interacts with, supports and creates value in conjunction with other social and cultural systems (see Table 1). SS research in agriculture can contribute to the five streams in the SSR program, which I will now introduce.

### Five streams of the SSR program in marketing

#### Stream 1: Build functional, integrated definitions

The focus of the first stream is developing functional, integrated definitions for sustainability. This means building from issues that have been flagged with past SA research to create actionable frameworks that can lead to measurable environmental and social improvements (Milne et al. 2006). This is reflected in epistemologies that allow for a holistic examination of production and consumption activities and associated externalities across supply chain locations (Gray 2010; Higgins et al. 2018). What common elements in SA exist across different instantiations and projects? What goals are communicated by producers, consumers and marketing agents across SA firms in different contexts? Several articles explore reasons actors engage in SA and identify specific associated rhetoric (Press and Arnould 2011a and b; 2014; Thompson and Coskuner-Balli 2007; Fitz-Koch et al. 2018; Weber et al. 2008). Future research could address how individual actors’ goals shape local markets (Mars and Schau 2017, 2018; Visconti et al. 2014) and how SA producers and consumers identify, experience, interpret and manage changing markets and marketing strategy. Investigations could also address how SA takes a variety of stakeholders (NGOs, different social groups; entrepreneurs; policy makers; corporate agricultural players; natural ecosystems) into account in exchange models and value creation. What can we learn from different instantiations of SA in the past 80 years? For example, what can previous SA projects tell us about the interaction between exchange and production models and consumer behaviors and interpretations? Finally, how do SA producers interact with agricultural firms and local businesses, and how could they work with local institutions to increase capacity around SA (Sen 2005)? Exploring these questions will build a contextualized understanding of SA across stakeholders. Such an understanding is key to identifying who and what is included and excluded in current instantiations of SA, where and at what level the focus of SA projects has been.

#### Stream 2: Explore the limits of existing research

This stream explores past research on the agriculture industry, broadly speaking, and the ways that sustainability has (not) been taken into account. This involves looking at the assumptions, goals and research questions in past research to see whether and how such studies could be reimagined as SS studies (see Tables 1 and 2). Research in this stream could explore how agricultural firms understand and manage the effects of the climate crisis and how firm operations and business activity would need to change to operate within ecological limits (Hobson 2013) and to build systemic interdependence (Sen 2005). What can past work on SA tell us about how managers use their knowledge of consumers to increase innovation and build their brand (Cayla and Arnould 2013; Etimur and Coskuner-Balli 2015)? What kind of consumer position is assumed and created in previous SA research (Dolan 2002)? For example, what can we learn from the different ways that CSA vs. the Slow Food movement vs. dumpster divers (Gollnhofer et al. 2019) identify the consumer position? What do we know about where value creation in SA ventures is (not) directed (Karababa and Kjeldgaard 2014; Porter and Kramer 2011)? Further, the body of MMR on sustainable consumer behaviors around food choices could be analyzed as a whole (c.f. Armstrong Soule and Reich 2015; Winterich et al. 2019; Luchs et al. 2010; Karmarkar and Bollinger 2015; White and Simpson 2013) to identify what we already know about food, consumer choice and marketing strategy, as they pertain to sustainability, when we look across studies that address a variety of micro-level phenomena. Such a meta-analysis could help define the landscape of our knowledge and identify areas where knowledge of micro-level phenomena could be integrated into a broader cultural perspective. Further, what could big data tell us about SA in terms of consumer preferences and industry practices? Finally, we could identify the ways that past SA research cannot and does not contribute to strong sustainability whether through assumptions about stakeholders and exchange models, methodologies, levels of analysis or interpretation.

#### Stream 3: Create new models and frameworks

The third stream builds on the insights developed in testing the limits of current marketing knowledge (stream 2). Business model (BM) research in other fields is acknowledged as having the potential to incite systemic change (Bidmon and Knab 2018; Gambardella and McGahan, 2010; Johnson and Suskewicz 2009; Wells 2013). As such, BMs are seen as value creation mechanisms rather than operational frameworks (Bidmon and Knab 2018; Zott and Amit 2010; Press et al. 2020). Recent scholarship has identified BM innovation as a necessary part of societal transitions because they have “the potential to disrupt entire industries, because they connect multiple actors, [and] mediate between the production and the consumption side of business” (Bidmon and Knab 2018, 903).

Herbert et al. (2018) show the urgency for food retailers to find new marketing strategies due to increasing pressures in the upstream supply chain and changing consumer preferences. They identify a change in large retailers’ stance toward SA, from seeing local producers as competition to be smothered, to seeing them as potential collaborators and even reaching out to small producers to start a dialogue around creating a robust local food system. Sebastiani et al. (2013) examine the collaboration between a company and a social movement to develop a new business. Their research highlights how two different actors aligned and communicated values and goals in marketing and exchange experiences. Thus, research in this stream could explore alternative SA models and what they could mean for large-scale sustainability. What concerns and constraints around sustainability do existing models and experiments expose in terms of social justice and economic issues (Gottschlich and Bellina 2017)?

Research in this stream could look at how other fields (e.g. social movements, societal transitions) have conceptualized SA and explore how those models could be used to inform our conceptualization of customers, firms, markets and exchange in the agricultural industry. What innovations could be taken from other collaborative models we currently know about (e.g. sharing and access platforms and enhanced crowd-funding)? How do different SA models account for the effects of strict limits to growth (as defined by the natural ecosystem) and economic incentives to engage in sustainable practices (e.g. payment for the full cost of ecological damages)? From a different perspective, what could event models of, for example, SA projects within a city (c.f. programs in Detroit; Michigan Urban Farming Initiative n.d.; Detroit Food Policy Council (DFPC) n.d.) show us about the integration of SA with other municipal projects and social systems? How would models of SA projects change our understanding of their goals, achievements, blind spots and value creation? How could modeling SA projects contribute to new SA innovation?

#### Stream 4: Build interdependent research streams

The fourth stream highlights the need to integrate levels of analysis from micro to macro, from the individual unit to the ecosystem, across time, into dynamic research streams. This stream responds to calls for such integrated and contextually-situated work (Askegaard and Linnet 2011; Fitchett et al. 2014; Thompson et al. 2013; Moisander et al. 2009; Earley 2014; Fitchett et al. 2014). It brings in broad stakeholders including industry players, NGOs, institutions, governmental bodies and additional supply chain players that reflect contextual influences on consumers and markets, thus recognizing that markets are complex, performative, changing social entities (Araujo 2007; Kjellberg and Helgesson 2007) and need to be explored and understood through additional points of entry.

Here, scholars explore the macro context for individual consumption practices (Dolan 2002) and look at the relationship between consuming and conserving resources (Campbell et al. 2013). An example of this can be seen in a paper by D'Antone and Spencer (2015)), in which they take a holistic approach to examining sustainable palm oil consumption. They identify this consumption as a market-wide issue, rather than one relating to individual consumers or actors in the supply chain. Further, they introduce the idea of multiplex consumption, which embraces an agency among a variety of stakeholders (consumers and non-consumers) and “ideas, representations, devices and metrics, in interaction, and where the sum effect for consumption across the market is greater than the sum of its parts” (68). Scholars might also explore the interplay among historic trends (Press and Arnould 2011b) and entrepreneurial, eco-entrepreneurial and social entrepreneurial activity among consumers (Stål and Bonnedahl 2016; Thompson and Coskuner-Balli 2007). The relationship between consumption and delivering value could be deepened through collaboration between service dominant logic (SDL) and CCT researchers (Arnould 2007; Campbell et al. 2013). For example, what would a consumer-centric view of SA look like (Arnould 2008) and how could that shape entrepreneurial and industrial activity?

This stream could further explore value creation and distribution in terms of co-evolving interdependencies, as the French grocery retail industry is doing with local food purveyors (Herbert et al. 2018; Dresner 2002; Gray 2010; Lenton et al. 2019; Morrison et al. 2019). We could use our research insights to facilitate collaborations across social movements, enterprise and entrepreneurs (Herbert et al. 2018; Sebastiani et al. 2013), to leverage increases in SA and integration of SA projects into the activities of other institutions, to integrate sustainable consumption efforts with production and supply chain constraints (D'Antone and Spencer 2015), and to identify ways that business and society integrate for greater human and environmental health and social justice.

Finally, research in this stream encourages critical reflexivity (Ger 2018) in our approach to SA research and in the clarification of our goals (Flyvbjerg 2005), seeking to make explicit the legacy of our own history (Cova et al. 2013), embedded ideologies and myths about agricultural production, marketing (Press et al. 2014; Thompson and Press 2014), markets and exchange practices (Visconti et al. 2014). Scholars should explore the structural, institutional, and political factors shaping particular system dynamics and informing and influencing power relations and privileged positions (Ger 2018).

#### Stream 5: Focus on impact

The final research stream focuses on impact. MacInnis et al. (2020) point out that marketing can do a better job of producing research that is relevant to multiple stakeholders outside academic circles, including industrial actors and policy makers. They suggest that authors anchor their research program with a substantive issue; this could be sustainability. The agriculture industry faces substantive concerns about remaining viable with changing demand, unpredictable supply and decreasing stomach for negative externalities. Sustainability is a place where marketing researchers could contribute to the development of new business strategy and policy creation by building links to share our research findings with thought leaders. The transformative consumer research (TCR) group has put efforts towards socially impactful research for many years. In a new effort to connect academic research to policy makers and industrial actors the Academy of Consumer Culture, Equitability, and Sustainability Studies (ACCESS n.d.) was recently created to connect CCT-research insights about consumers and consumption with policy makers, to inform public debate, and identify answers to challenges related to sustainability and the climate crisis (see https://getaccessnow.eu/). If we undertake SSR as a group effort, there is much more marketing academia can do to shape industrial, political, and social conversations and actions around sustainability.

I have used SA as the context to illustrate the SSR program I have set out. I now offer two additional brief industrial examples of where an SSR program could be applied. First, the fashion industry is currently dominated by a fast fashion business model, which is based on the rapid introduction of low quality, low-priced ready-to-wear items that often copy high end brands. This business model is criticized for its human rights abuses and negative environmental externalities (Fletcher 2008; McRobbie 1997; Morgan and Birtwistle 2009; Ozdamar Ertekin and Atik 2015), which are made worse by the ever-increasing volume of clothing consumption (Goworek 2011; Gam et al. 2009; Defra 2010). The textile industry is one of the most polluting sectors in the world (Cruz et al. 2017). Due to massive backlash from NGOs and consumers, and new governmental policies (Samuel 2019), firms are required to develop new marketing strategies and business models. Fast fashion firms are struggling to identify how to build strategy for a market (Pedersen et al. 2015) that is collapsing from the supply side and the demand side (Grosclaude 2019). Fast fashion firms are grasping at straws, adopting uninformed temporary strategies, and looking to sustainable fashion start-ups for ideas on how to proceed (e.g. Plateau Fertile; Fashion Green Days n.d.). The fashion industry needs insight into how to keep their brand identity while radically shifting their business models (Holt 2004; Press et al. 2020). In addition, it needs new customer and product approaches, which could be led by entrepreneurs who identify specific needs in localized communities (Scaraboto and Fischer 2013). SSR for the fashion industry could also follow previous CCT (D'Antone and Spencer 2015) and critical research (Dolan 2002) examples that build insight into contextual issues affecting activity in every part of the supply chain.

Second, the steel industry runs on high energy consumption and high carbon emissions, making it vulnerable to pressures to relocate and shut down (Rynikiewicz 2008). The steel industry has worked to reduce carbon emissions, and has opportunities to continue in that direction (Johansson and Söderström 2011). In a collaboration with the national gas company, the ArcelorMittal plant in Dunkirk, France built a combined cycle gas-powered power plant that recovers an estimated 5 billion m^3^ of steel production gases. Spurred by social pressure, ArcelorMittal began to engage with local NGOs, civic groups, industrial partners and municipalities to measure and report air quality and to sponsor local entrepreneurial projects (Press et al. 2020). The Dunkirk steel plant has become a hub of entrepreneurial activity that exploits the energy and waste flows from the plant, housing a cement company, a company that recovers precious metals, and a hydrogen conversion plant. The integration of this steel plant into the local sustainability culture has taken many years, and continues to be contentious. However, ArcelorMittal struggles to innovate strategies for creating value across multiple stakeholders (Press et al. 2020), and has not identified models or strategies for operating with increased pressure on them to change their practices. SSR for the steel industry could also address these multi-stakeholder issues from the perspective of a united ecosystem, building insight into tensions among economic issues, social issues and issues of overall health of humans, animals and nature.

## What it means for marketing researchers to work on an SSR program

I join the decades of calls for expanding the scope and relevance of marketing research, citing its focus on micro-level constructs and increasingly sophisticated methodologies (Hunt 2018; Houston 2016; Varadarajan 2010, 2015, 2018), and lack of effort to integrate and extend its reach (Biggadike 1981; Hunt 2018), leading to a shrinking sphere of influence (Reibstein et al. 2009; Clark et al. 2014). MacInnis et al. (2020) present a model of necessary elements for producing relevant and impactful research. Their suggestions push researchers beyond the tacitly accepted boundaries of marketing research toward a more systemic, networked, holistic approach (Moorman et al. 2019) to identifying research questions, and designing and executing studies. SSR is a domain of relevance and urgency that breaks implicit boundaries in research design and execution (MacInnis et al. 2020). It could help revitalize MMR (Biggadike 1981; Hunt 2018; Houston 2016; Varadarajan 2010, 2015, 2018; Reibstein et al. 2009) by identifying more meaningful research questions (Moorman 2016) that can address industry-relevant needs by building new marketing strategy and identifying new business models (Press et al. 2020), exchange methods (Cova et al. 2013), and ways of performing markets (Araujo 2007).

While CCT does a better job at including in-situ respondents engaging in the activities under study (MacInnis et al. 2020), this point is worth exploring when it comes to SSR. Because SSR is situated in substantive and immediate industrial needs, we have the benefit of being able to talk with individual marketing managers, as we are accustomed to do. However, individual managers should be identified as starting points (not ending points) from which to identify, investigate, and interact with other stakeholders, including NGOs and government organizations, communities impacted by firm and industry operations, and the natural environment (e.g. SDGs). Further, as critical marketing scholars have identified, the broader context within which managers act, including the nonmarket systems and associated cultural values that support market exchange (Sen 2001), must be taken as interdependent with firm activities.

True collaboration across CCT and MMR approaches has been elusive. As researchers, we may agree on axiology, such as the value of researching sustainability in business, however, we have ontological differences that have prevented us from working together, and which may be stifling our progression as a field. Strong sustainability provides a common ontological platform from which to build collaboration across approaches (see Table 2). The SSR program provides a framework built around SS principles, and articulates assumptions about value creation and exchange, the interdependent nature of systems, and the relationships among history, society and resources. SSR thus provides a context in which epistemological differences across approaches can become complementary resources in collaborative projects, rather than barriers to working together, and facilitates integration of our own traditions and dialogue with neighboring disciplines (Tadajewski 2018a; Hunt 2018). The five SSR streams identify areas for inquiry and the sustainable agriculture example illustrates how these research streams can be applied to substantive issues faced by a particular industry (MacInnis et al. 2020). My hope is that all researchers interested in, or curious about contributing to SSR see a place for themselves in each research stream, and that researchers use the SSR-focused conceptualization in Table 2 to build their projects. In this way, we may find a domain for collaboration that allows us to harness the full power of our collective intellectual and methodological strengths.

I am not suggesting that the field of marketing abandon, or even discount, past research. However, I am suggesting that we have a responsibility to take a directed and vocal stand about sustainability issues. We have an opportunity to inform policy and develop new marketing strategies for firms facing new industrial contexts that are explicitly situated within the limits of nature—what is ecologically possible (Hobson 2013).

## Concluding remarks

Past research on sustainability in marketing has largely addressed micro-level issues in a WS context. However, many industries and communities are grappling with SS issues. That is, they are facing issues that fundamentally question whether they can exist in the current growth-based capitalist system, and they are exploring other options, some of which may have been scoffed at a few years earlier (Herbert et al. 2018). The five research streams I identify mark a path forward for CCT and MMR researchers to begin addressing sustainability issues in a concerted effort, for maximal impact.

Given the complex and integrated nature of sustainability, it must be explored through transdisciplinary (Brown et al. 2010; Crane and Desmond 2002; McDonagh and Prothero 2014; c.f. Martin et al. 2019) and multi-level approaches. Insular and micro-level research cannot address strategic industry needs in an SS context. Further, micro-level research cannot guide the development of specific pieces of marketing strategy, such as innovation, branding, consumer and social value creation or market orientation, nor can it identify insights into interconnected and ecosystemic issues. A CCT perspective is able to integrate micro-, meso- and macro-levels of analysis (Giesler 2003, 2008; Giesler and Fischer 2017) as well as lived experience and explanations for the conditions of the experiences (Arnould and Thompson 2005; Arnould et al. 2019), and broader contextualization for those experiences, which may include “structuring influences of market and social systems that is not necessarily felt or experienced by consumers in their daily lives, and therefore not necessarily discursively expressed” (Askegaard and Linnet 2011, 381; Moisander et al. 2009; Earley 2014; Fitchett et al. 2014).

There is a bolder and more ambiguous opportunity here as well, which is to emerge as thought leaders on sustainable business practices, market operations and new exchange and consumption models, developing theory around crucial issues in business strategy in rapidly changing markets (Arnould et al. 2019). Thus far, no business discipline has emerged as a leader in SSR. While there are many books and articles on sustainability topics, they largely promote WS and fail to address the industrial pressures driving a need for more radical strategy change, from brand management to customer relations to innovation.

Finally, for some researchers, exploring strong sustainability may seem like writing science fiction, but we must remember the impact science fiction has had on innovation. This path forward will bring internal meaning and external interest to our field, and it could also change the way we conceptualize exchange, identify the purpose of business, and integrate industry and society; it could change the world.
